# Phenolic Biotransformations during Conversion of Ferulic Acid to Vanillin by Lactic Acid Bacteria

**DOI:** 10.1155/2013/590359

**Published:** 2013-08-28

**Authors:** Baljinder Kaur, Debkumar Chakraborty, Balvir Kumar

**Affiliations:** Department of Biotechnology, Punjabi University, Patiala 147002, India

## Abstract

Vanillin is widely used as food additive and as a masking agent in various pharmaceutical formulations. Ferulic acid is an important precursor of vanillin that is available in abundance in cell walls of cereals like wheat, corn, and rice. Phenolic biotransformations can occur during growth of lactic acid bacteria (LAB), and their production can be made feasible using specialized LAB strains that have been reported to produce ferulic acid esterases. The present study aimed at screening a panel of LAB isolates for their ability to release phenolics from agrowaste materials like rice bran and their biotransformation to industrially important compounds such as ferulic acid, 4-ethyl phenol, vanillic acid, vanillin, and vanillyl alcohol. Bacterial isolates were evaluated using ferulic acid esterase, ferulic acid decarboxylase, and vanillin dehydrogenase assays. This work highlights the importance of lactic acid bacteria in phenolic biotransformations for the development of food grade flavours and additives.

## 1. Introduction

Vanillin is the most widely used flavor compound in the food industry. Because of the scarcity and higher cost of natural vanilla extract, there has long been interest in the biotechnological preparation of vanillin. The most important way has been investigated by implementing ferulic acid esterase (FAE) producing microbes to obtain ferulic acid (FA) from agrowastes like wheat bran [1] and rice bran [2]. FAE producers hydrolyze sugar-phenolic acid ester linkages present in cell wall and assimilate FA as a sole carbon source, which can also be used as the most promising process for vanillin production by biotransformation [3]. Degradation of FA to other metabolic products like 4-vinyl guaiacol (4-VG) and 4-ethyl phenol (4-EP) reported previously in many strains of lactic acid bacteria (LAB) by decarboxylase enzyme is a major obstacle in their industrial implementation [4]. Moreover, vanillin which is a phenolic aldehyde shows toxic effect on the microbes and gets reduced spontaneously to less toxic intermediates like alcohol (vanillyl alcohol) or acid (vanillic acid). This metabolic degradation could interfere with vanillin production by reducing its amount in the biotransforming media, which has also been previously reported by many scientists [5].

Various metabolic pathways have been described for the production of vanillin from various organic substrates but there is a very little evidence for significant vanillin production using LAB so far [6]. It has also been reported that nonoxidative decarboxylation followed by reduction is responsible for degradation of phenolic compounds in many LAB especially *Lactobacillus*, where production of 4-VG and 4-EP with traces of vanillin has been reported [4, 7]. LAB isolates from wine like *Oenococcus oeni*, *Lactobacillus brevis*, *L. hilgardii*, *L. plantarum*, and *Pediococcus damnosus* also have phenolic transformation properties. It was reported that *Oenococcus oeni* or other *Lactobacillus *spp. facilitate malolactic fermentation during wine production and can improve wine aroma by degrading phenolic acids [8]. It has also been reported that *Oenococcus *sp. were not able to convert vanillic acid (VA), eugenol, or isoeugenol into vanillin, but can degrade FA into a very little amount (1%) of vanillin which gets further reduced to vanillyl alcohol (VAL). Production of high yields of 4-VG from FA by nonoxidative decarboxylation for the first time was reported in *L. farciminis* [8, 9]. In the present study, panels of LAB isolates were screened for their ability to release FA, and other phenolic acids from agrowaste material like rice bran and their biotransformation to phenolic compounds such as FA, vanillin and other phenolic derivatives like 4-EP, VA, VAL, and so forth. 

## 2. Materials and Methods

### 2.1. Chemicals

FA (99% pure), VAL (99%), and 4-EP (97%) (Fisher Scientific), vanillin (99%), and hydrochloric acid (35–38%) (S.D. Fine-Chem. Ltd.), ammonium nitrate (98%, Nice Chemicals Pvt. Ltd.), maltose (98%, Sisco Research Laboratories Pvt. Ltd.), thiobarbituric acid (99%, BDH), VA (98%), dextrose (99%), tri-ammonium citrate (98.5%), di-potassium hydrogen phosphate (99%), magnesium sulphate (99.5–100%), manganous sulphate (98%), sodium acetate (82.03%), sodium dihydrogen orthophosphate (99%), disodium hydrogen orthophosphate (99%) (HiMedia Laboratories Pvt. Ltd.) were obtained and were used for HPLC analysis and enzymatic assays.

### 2.2. Microorganisms and Culture Conditions

Nine LAB isolates 16, 18, 20, C1L, C1S, P2 (25), P2, GML, and V1 were evaluated in this study, isolated by enrichment procedure on MRS medium (20 g L^−1^ dextrose, 10 g L^−1^ peptone, 10 g L^−1^ beef extract, 5 g L^−1^ yeast extract, 5 g L^−1^ sodium acetate, 2 g L^−1^ tri ammonium citrate, 2 g L^−1^ dipotassium hydrogen phosphate, 0.1 g L^−1^ magnesium sulphate, 0.05 g L^−1^ manganous sulphate, and 1 mL tween-80; pH 5.6) at 37°C for 24 h in 250 mL flasks. After two subculturing twice, 1% inoculum was transferred into 250 mL Erlenmeyer flasks containing 50 mL of MRS medium and then cultivated overnight and further used for enzymatic assays.

### 2.3. FAE Activity Assay

Extracellular FAE activity was determined according to the method previously described by Kaur et al. [2] with some modifications in rice bran medium (100 g L^−1^ rice bran, 2.4 g L^−1^ maltose, 1 mL tween-80, pH-5.6) at 37°C for 24 h, using 1% v/v inoculum. The released FA was extracted with equal volume of ethyl acetate and was determined from the optical density (OD) at 310 nm using a FA standard curve. The specific activity was calculated as nkatals mg^−1^ protein. One nkatal (nanokatal) is defined as the amount of enzyme that catalyzes release of 1 nM of free FA per second [10].

### 2.4. Ferulic Acid Decarboxylase (FDC) Activity Assay

Cells were harvested by centrifugation and were resuspended in 70 mM sodium phosphate buffer (pH 6.0) containing 0.1 g L^−1^ FA and incubated at 30°C for 8 h. Samples were centrifuged hourly and supernatants were kept on ice prior to analysis. FDC activity was determined within the UV range of 250 to 350 nm according to standard methodology as described previously by Kaur et al. [2].

### 2.5. Vanillin Dehydrogenase (VDH) Activity Assay

VDH activity was determined according to Converti's protocol [11] with some modifications. Vanillin synthetic medium consisted of 1 g L^−1^ vanillin, 1 mL tween-80, 2 g L^−1^ triammonium citrate, 5 g L^−1^ sodium acetate, 0.1 g L^−1^ magnesium sulphate, 0.05 g L^−1^ manganous sulphate, and 2 g L^−1^ di-potassium hydrogen phosphate. The VDH assay was carried out at pH 5.6 and with vanillin was used as sole carbon source. The best isolate (P2) was cultured in 500 mL flasks containing 100 mL vanillin synthetic medium at 37°C for 24 h. 1 mL supernatant from the synthetic medium was withdrawn after every 8 h intervals and centrifuged. The supernatant was mixed with 5 mL of 24% HCl solution and 2 mL of 1% thiobarbituric acid in a test tube. It was heated in a 55°C water bath for 10 min and subsequently stored at room temperature for 20 min. The decrease in absorbance was then recorded at 434 nm. 

### 2.6. Biotransformation of Rice Bran Phenolics

Isolate P2 possessing negligible VDH activity was undertaken in this study, and biotransformation of rice bran to various phenolics was assayed in modified rice bran (RB) medium (150 g L^−1^ rice bran, 0.05 g L^−1^ ferulic acid, 5 g L^−1^ peptone, 0.005 g L^−1^ MgSO_4_, 2 g L^−1^ tri ammonium citrate, 2 g L^−1^ ammonium nitrate, 2.4 g L^−1^ maltose, and 1 mL tween-80; pH-5.6) at 37°C for 24 h, using 1% v/v inoculums [12].

### 2.7. Identification of Phenolic Metabolites by HPLC-UV, LCMS-ESI, and GC-MS

After every 8 h intervals, 2 mL aliquots from the biotransforming medium containing phenolic metabolites produced by the selected isolate P2 were partitioned with equal volumes of ethyl acetate. The ethyl acetate fraction was separated for HPLC analysis (Shimadzu, UV detector, column C-18, length-25 cm, and ID-4.6 mm) according to Landete et al. [13] and LCMS-ESI (Thermo Fisher Scientific, model: LTQ-XL, LCMS with ESI probe for direct injection mass) in the mass range of 100–300 [12]. Samples were filtered through a 0.45 *μ*m PVDP filter (pore size-0.2 micron, Durapore) and injected into the column as specified above. The solvent from the ethyl acetate fraction was evaporated using a rotary vacuum evaporator at 50°C and phenolic concentrates were recovered in 2 mL of 80% (v/v) methanol [14]. The boiling point of ethyl acetate is 77°C, and the extractions were performed at a much lower temperature that does not interfere with the physic-chemical properties of most of the phenolics tested in the study [15]. GC-MS (Shimadzu, Model: QP-500) was performed in DB5 column (length-30 m, ID-0.25 mm) in the mass range from 100 to 300 in order to confirm the presence of vanillin, other phenolic metabolites after complete extraction. FA, vanillin, VA, 4-EP, and VAL were used as standards.

### 2.8. Statistical Analysis of Results

One-way ANOVA analysis was carried out, and the results are presented as mean ± standard deviation of three triplicate experiments, and *P* value < 0.05 and *F* value > *F*-critical were taken as the criterion of significance.

## 3. Results and Discussion

### 3.1. FAE Assay

FA is present as ester-linked dehydrodimers in wheat bran, which can be released by FAE enzyme activity by *Streptomyces* sp. [16]. FAE of *S. avermitilis* CECT 3339 could release 10% FA by hydrolyzing destarched wheat bran [17]. In 1997, Faulds and his coworkers [18] reported that free FA is able to induce FAE activity in *Aspergillus niger* but not the esterified FA. Out of nine LAB isolates, three bacterial strains were selected on the basis of specific FAE activity (Table 1). Among different strains, maximum specific FAE activity of 0.294 ± 0.035 nkatals mg^−1^ was exhibited by the isolated strain C1L after 24 h culturing on RBM medium. It was followed by isolates GML (Gol Market Lassi—a buttermilk sample) and P2 with 0.273 ± 0.05 and 0.190 ± 0.015 nkatals mg^−1^, respectively. Maximum FAE activity was observed after 24 h as compared with 96 h incubation required in case of *Streptomyces* sp. S10 [16], 48 h in *Streptomyces* sp. [17], and 72 h in another *Streptomyces* sp. [19]. 

### 3.2. FDC Assay

**Figure 1:**
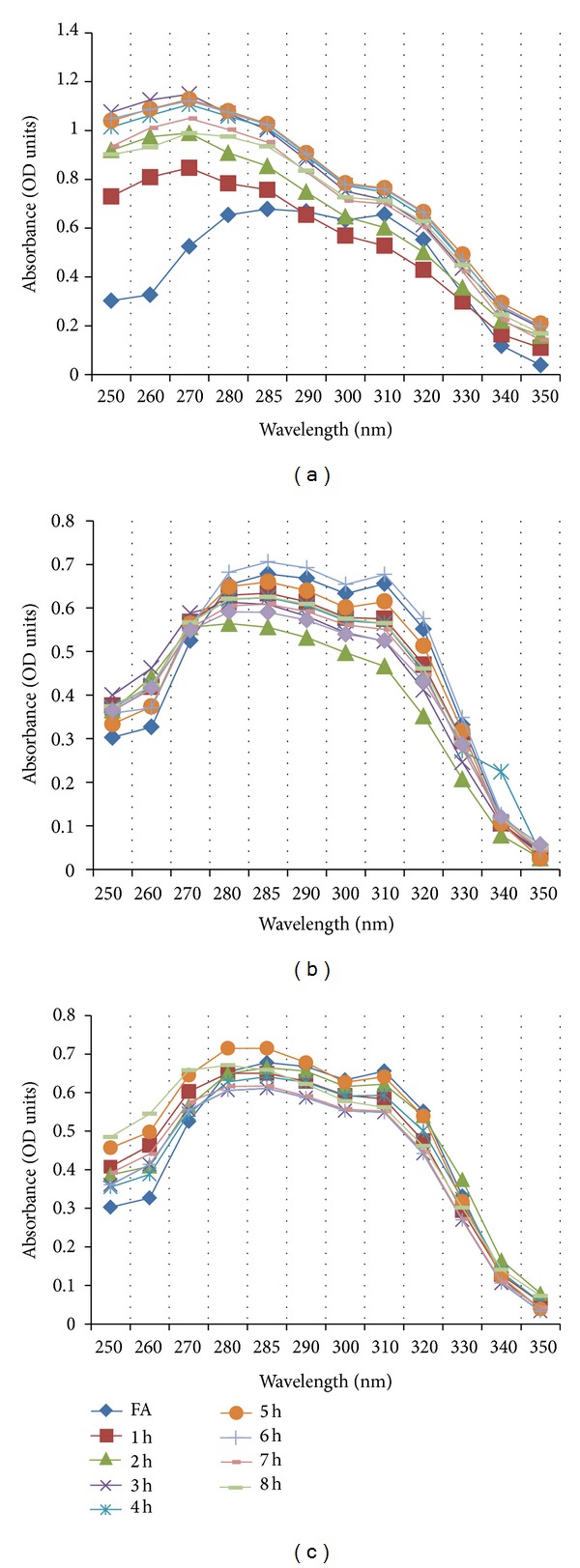
FDC plots of selected lactic acid bacterial isolates; (a) C1L, (b) GML, and (c) P2.

C1L, GML, and P2 were implemented in this study; consequently, P2 was found to be the best isolate as it is the least FA degrading as observed by minimum fall in absorbance between 285 and 310 nm (Figure 1). Decarboxylase activity of C1L and GML isolates degrades ferulic acid rapidly where P2 isolate retains it. Decarboxylation of FA has already been described for several LAB including *Pediococcus* sp. [2] and *Lactobacillus* sp. [20]. In most cases, the conversion of FA to 4-VG by nonoxidative decarboxylation has been described [7, 9]. In *B. coagulans* BK07 FA is rapidly degraded into 4-VG by nonoxidative decarboxylation and is then converted to vanillin within 5-6 h of growth. After 7-8 h, VA along with trace amounts of protocatechuic acid was detected in the culture extract due to oxidation and followed by demethylation, respectively [21]. 

### 3.3. VDH Assay

**Figure 2:**
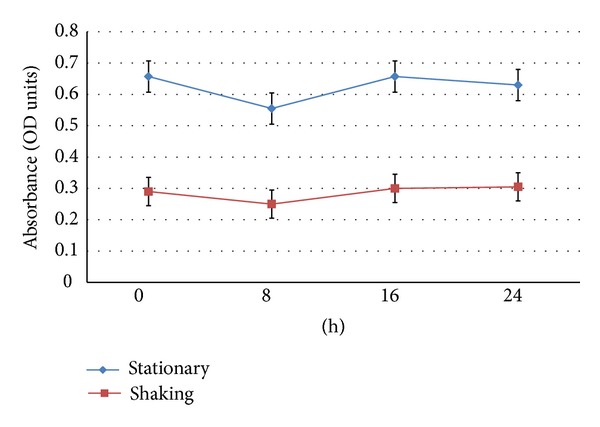
VDH plot of (P2) isolate.

Isolate P2 does not degrade vanillin as evidenced by its VDH negative property. The concentration of vanillin was constant throughout the assay and throughout the study as absorption of vanillin remained unchanged at 434 nm except at 8 h (Figure 2). This might be due to the consumption of vanillin by the P2 isolate and its conversion into vanillyl alcohol and vanillic acid during 8th hour of the growth, which was later replenished through some unknown vanillin biosynthesis pathway of the P2 isolate. Other phenolics such as VAL or VA are produced during phenolic biotransformations using LAB [2, 7, 9] and other microbes [21, 22] which can utilize FA as their carbon and energy source. Separate experiments have indicated that isolate P2 can tolerate high concentrations of FA and vanillin, thus would be an excellent host for the high level production of vanillin and other phenolics by introducing metabolic pathway which was previously reported in *P. fluorescens* strain vdh-BF13 strain [22].

### 3.4. Biotransformation of Rice Bran to Phenolic Metabolites and Their Detection by HPLC-UV, LCMS-ESI, and GC-MS

**Figure 3:**
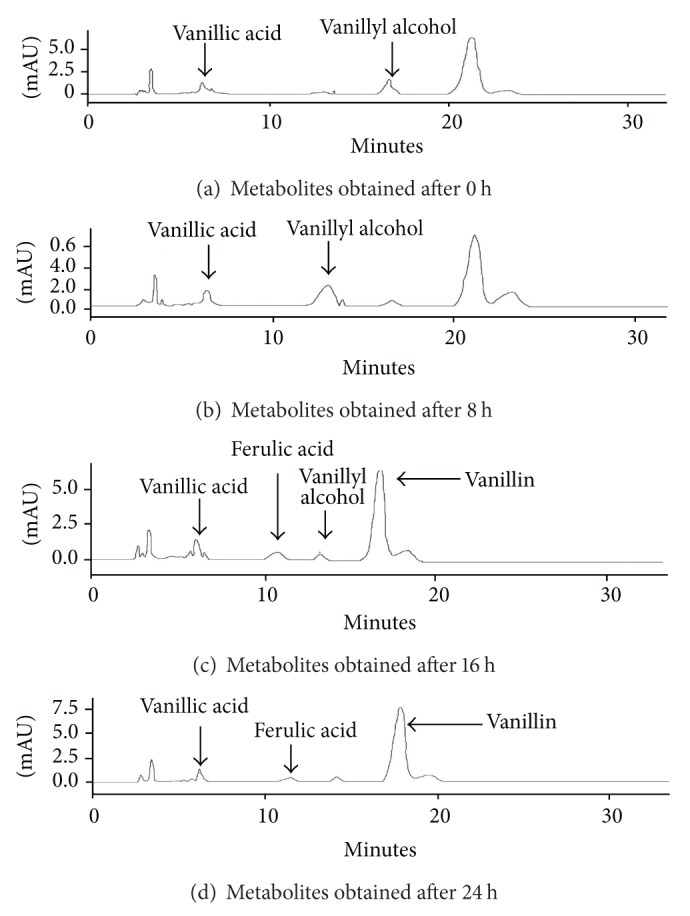
Detection of phenolic derivatives by HPLC-UV analysis.

**Figure 4:**
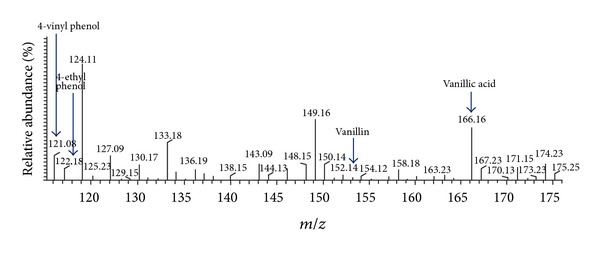
Confirmation of vanillin and other phenolic metabolites by LCMS-ESI.

**Figure 5:**
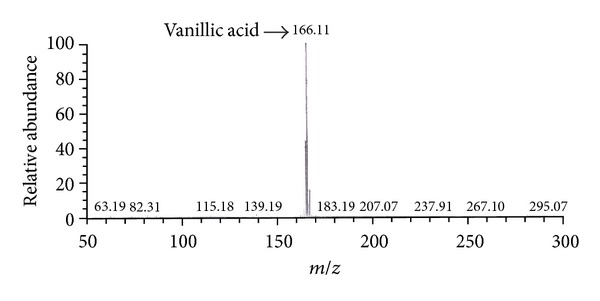
Confirmation of vanillic acid by GC-MS.

Ethyl acetate extractions were performed to separate phenolic metabolic products from the biotransforming media which were finally detected using HPLC-UV and LCMS-ESI. Occurrence of vanillin, VAL, VA, FA, and 4-EP metabolic intermediates was reported during biotransformation of rice bran to phenolics by P2 isolate. Initially at 0 h and 8 h, VA and VAL were detected in the medium but with increasing time at 16 h and 24 h; vanillin and FA were also detected, which confirms vanillin production in the rice bran media at 16 h of biotransformation (Table 2 and Figure 3). LCMS-ESI chromatogram also confirms the presence of vanillin; VA and 4-EP as a result of phenolic biotransformation mediated through LAB isolate P2 on modified RBM (Figure 4). Repeated extractions led to production of vanillic acid which was confirmed by gas chromatography (Figure 5). This work presents the inherent capacity of isolate P2 to decarboxylate the naturally occurring FA for production of natural vanillin. Sugar beet pulp and maize bran have been previously used as a carbon source for FA production using *A. niger* I-1472, which was then further converted to VA for the production of natural vanillin [23].

FA is a readily available natural raw material for the biotransformation to vanillin using biocatalysis. The biodegradative metabolic pathways of FA have been reported in many microorganisms including LAB [2, 7, 8, 21]. In *Sporotrichum thermophile* strain, FA is degraded via the propenoic chain into 4-VG, which was presumably metabolized to VA and it is finally converted to guaiacol by nonoxidative decarboxylation as described by Topakas and his workers [24]. The p-Coumaric acid decarboxylase (PDC), which plays a major role in the degradation of phenolic acid such as p-coumaric, m-coumaric, caffeic, ferulic, gallic, and protocatechuic acid has been overexpressed and purified from *Lactobacillus plantarum* CECT 748(T). PDC catalyzes the formation of the corresponding 4-vinyl derivatives, that is, vinylphenol and vinylguaiacol from p-coumaric and FA, respectively, that have been approved as flavoring agents [20]. 

## 4. Conclusion

C1L, GML, and P2 isolates were selected from FAE assay and implemented in FDC assay. Out of all three, the P2 isolate showed maximum FA retention property and was further employed in the VDH assay. Finally, phenolic transformation and production of vanillin, FA, VAL, VA, 4-EP, and so forth, by the isolate P2 in rice bran media, were studied. Agro byproducts such as rice bran, which is produced in more than 10,000,000 tons per year in the rice refining industry, contain abundant amount of FA [25]. Considering the escalating demand for natural flavors in the food industry, this work focused on the production of important vanillin precursors by* Pediococcus acidilactici* isolate P2 in rice bran media, which has not been previously reported. Several authors have reported that DSWB is the main carbon source for FAE production by *Streptomyces* [17, 26, 27]. However, in this study, FA supplemented RB medium was found as most suitable medium for vanillin production. Vanillin with 4-EP (product of FA decarboxylation), VAL (obtained after vanillin degradation), and VA were detected in the medium but the rate of conversion to these products from FA and vanillin was found to be very slow. This may either be a result of C_1_/C_2_-cleavage of FA side chain or due to cleavage of vinyl bond of 4-vinyl guaiacol, which is coproduced in the modified RBM medium during LAB biotransformations. 

## Figures and Tables

**Table 1:** FAE activity of selected LAB isolates.

Strain name	Specific activity (nkatal/mg)
16	0.173 ± 0.02
18	0.072 ± 0.01
20	0.118 ± 0.016
C1L	**0.274 ± 0.035**
C1S	0.130 ± 0.005
P2 (25)	0.170 ± 0.018
P2	**0.190 ± 0.015**
GML	**0.273 ± 0.05**
V1	0.153 ± 0.04

*P* value < 0.05; *F* value > *F*-crit.

**Table 2:** Detection of phenolic metabolites on modified rice bran media by HPLC-UV.

	Ferulic acid	Vanillin	Vanillic acid	Vanillyl alcohol
0 h	—	—	3.60%	2.61%
8 h	—	—	3.58%	**17.21%**
16 h	6.20%	**5.01%**	**7.43%**	3.20%
24 h	—	4%	4.55%	—

Retention time (min): Vanillic acid—6.7, Ferulic acid—11.86, Vanillyl alcohol—13.1, Vanillin—18.9.
